# Comparative Effectiveness of Chlorhexidine-Alcohol and Povidone-Iodine in Preventing Surgical Site Infections: A Systematic Review and Meta-Analysis

**DOI:** 10.7759/cureus.104364

**Published:** 2026-02-27

**Authors:** Xiuli Feng, Jingli Dou, Yufang Zhu, Lin Ba

**Affiliations:** 1 Operating Room/Nursing, Binzhou People's Hospital, Binzhou, CHN; 2 Operating Room/Nursing, Binzhou Medical University Hospital, Binzhou, CHN; 3 Anesthesiology, Binzhou People's Hospital, Binzhou, CHN

**Keywords:** antiseptic, chlorhexidine-alcohol, clean-contaminated surgery, meta-analysis, povidone-iodine, surgical site infection, systematic review

## Abstract

Surgical site infections (SSIs) remain a leading cause of postoperative morbidity. While both chlorhexidine-alcohol (CHG-A) and povidone-iodine (PVI) are standard preoperative skin antiseptics, their comparative efficacy, particularly in clean-contaminated surgeries, where the benefit of CHG-A’s persistent activity is most theorized, remains a subject of ongoing clinical debate. This meta-analysis aimed to evaluate whether CHG-A is superior to PVI in preventing SSIs in adult patients undergoing elective surgery, with a specific focus on clean-contaminated procedures. The primary outcome was overall SSI incidence within 30 days; secondary outcomes included deep incisional SSI, superficial incisional SSI, and adverse skin reactions. We conducted a systematic review following the Preferred Reporting Items for Systematic reviews and Meta-Analyses (PRISMA) 2020 guidelines. PubMed, Web of Science, EMBASE, and the Cochrane Library were searched from inception to January 31, 2026, without language restrictions. We included randomized controlled trials (RCTs) comparing CHG-A with PVI for preoperative skin preparation in adults undergoing elective surgery and reporting 30-day SSI rates using standardized definitions. Two reviewers independently screened studies, extracted data, and assessed risk of bias using the Cochrane RoB 2 tool. A random-effects model (DerSimonian-Laird method) was used for the meta-analysis, with heterogeneity quantified using the I² statistic. A prespecified subgroup analysis was conducted for clean-contaminated surgeries, and a leave-one-out sensitivity analysis was performed. Nine RCTs (n = 8,000 patients) were included. For overall SSI, no significant difference was found between CHG-A and PVI (risk ratio (RR) = 0.84, 95% CI: 0.66-1.07; I² = 48%). In a prespecified subgroup of five studies on clean-contaminated surgeries, the RR was 0.77 (95% CI: 0.58-1.03). Deep incisional SSI rates were lower with CHG-A, although not statistically significant (RR = 0.75, 95% CI: 0.53-1.07). Heterogeneity stemmed from variability in surgery types and antiseptic formulations. Sensitivity analysis indicated robustness, with the most substantial effect change occurring upon omission of a large cardiac surgery trial. This meta-analysis did not find conclusive evidence that CHG-A is superior to PVI for preventing overall SSI across all surgery types. A nonsignificant trend favoring CHG-A was observed in clean-contaminated procedures and for deep SSIs, suggesting a context-dependent effect that warrants targeted investigation. Clinical choice may consider the specific surgical context, cost, and institutional protocols.

## Introduction and background

Surgical site infections (SSIs) are a prevalent health care-associated complication, contributing significantly to patient morbidity, extended hospital stays, and economic burden [1,2]. Preoperative skin antisepsis is a cornerstone of SSI prevention bundles [3].

Povidone-iodine (PVI), a broad-spectrum iodophor, has been a long-standing standard. Its efficacy, however, can be compromised by organic material, and it lacks persistent residual activity [4]. Chlorhexidine-alcohol (CHG-A) has emerged as a prominent alternative. Alcohol provides rapid, broad-spectrum killing, while chlorhexidine binds to the stratum corneum, offering sustained activity against microbial regrowth [5,6]. This persistent effect is theoretically most advantageous in procedures in which the surgical site is exposed to endogenous flora for prolonged periods, such as clean-contaminated surgeries (e.g., colorectal and hepatobiliary) [7].

While prior meta-analyses have suggested a benefit for CHG-A, their conclusions have been inconsistent and often limited by the inclusion of older or lower-quality studies [8,9]. Furthermore, several recent, large, high-quality randomized controlled trials (RCTs) have reported conflicting results, particularly in specific surgical contexts such as cardiac and cesarean delivery surgery [10-12]. This evolving evidence base creates uncertainty for clinical decision-making, especially regarding whether the putative superiority of CHG-A is universal or confined to specific surgical settings.

Therefore, this systematic review and meta-analysis aimed to provide a contemporary, rigorous synthesis of RCT evidence, with a focused hypothesis: In adult patients undergoing elective surgery, particularly clean-contaminated procedures, is preoperative skin preparation with CHG-A superior to PVI in reducing the risk of SSIs? The primary outcome was overall SSI incidence within 30 days postoperatively. Secondary outcomes included deep incisional SSI, superficial incisional SSI, and adverse skin reactions. We also sought to explore sources of heterogeneity and evaluate the safety profile of both agents.

## Review

Methods

This review was conducted and reported in accordance with the Preferred Reporting Items for Systematic reviews and Meta-Analyses (PRISMA) 2020 statement [13].

Study Design and Population, Intervention, Comparator, Outcomes (PICO) Framework

This systematic review was structured around the PICO framework to clarify the research question. The population (P) consisted of adult patients (aged ≥18 years) undergoing any elective surgical procedure. The intervention (I) was preoperative skin preparation with any formulation of CHG-A. The comparator (C) was preoperative skin preparation with any formulation of PVI, whether aqueous- or alcohol-based. The outcomes (O) included the primary outcome of overall SSI incidence within 30 days postoperatively and secondary outcomes encompassing the incidence of deep incisional SSI, superficial incisional SSI, and adverse skin reactions.

Eligibility Criteria

Inclusion criteria were (1) RCT design; (2) direct comparison of CHG-A vs. PVI for preoperative skin antisepsis; (3) adult elective surgical population; and (4) reporting of SSI outcomes using standardized definitions (e.g., CDC criteria) with a minimum follow-up of 30 days.

Exclusion criteria were (1) non-RCT designs; (2) studies involving emergency surgery or pediatric populations; (3) studies not reporting discrete SSI data for each group; and (4) studies in which the antiseptic intervention was part of a multifactorial bundle without isolated comparison.

Search Strategy

A systematic search was performed in PubMed, Web of Science, EMBASE, and the Cochrane Library from database inception to January 31, 2026. The search strategy combined terms for the intervention, comparator, outcome, and study design using Boolean operators. The core search logic was: ("Chlorhexidine-Alcohol" OR "Chlorhexidine" AND "Alcohol") AND ("Povidone-Iodine" OR "Iodine-based antiseptic") AND ("surgical site infections" OR "SSI") AND ("randomized controlled trial" OR "RCT"). This core structure was adapted as needed for each database’s syntax requirements. No language restrictions were initially applied, although only studies available in English full text were ultimately included. The reference lists of relevant reviews and included studies were manually screened. Full electronic search strings with database-specific adaptations are available from the corresponding author upon request.

Study Selection and Data Extraction

Two independent reviewers screened titles and abstracts and subsequently full-text articles against the eligibility criteria. Disagreements were resolved by consensus or consultation with a third reviewer. Data were extracted using a standardized, piloted form. Key extracted variables included first author, publication year, country, study design, sample size, patient characteristics, surgical specialty, wound class (clean vs. clean-contaminated), detailed intervention and comparator descriptions (specific formulation, concentration, volume, application method, and timing), SSI definitions, number of events per group for overall, superficial, and deep SSI, and reported adverse events.

Risk of Bias Assessment

The methodological quality of each included RCT was assessed independently by two reviewers using the revised Cochrane Risk of Bias tool for randomized trials (RoB 2), evaluating five domains: randomization process, deviations from intended interventions, missing outcome data, measurement of the outcome, and selection of the reported result [14].

Statistical Analysis

All analyses were performed using R software (version 4.3.x) with the* meta *and *metafor *packages. For dichotomous outcomes (SSI incidence), pooled risk ratios (RRs) with 95% CIs were calculated. A random-effects model (DerSimonian-Laird method) was applied a priori due to anticipated clinical heterogeneity. Heterogeneity was quantified using the I² statistic, with values of 25%, 50%, and 75% representing low, moderate, and high heterogeneity, respectively. Prespecified subgroup analyses were conducted based on surgical wound class (clean vs. clean-contaminated). Post hoc exploratory analyses examined the influence of specific surgical specialties. Publication bias was assessed visually using funnel plots and statistically using Egger’s regression test for the primary outcome. A sensitivity analysis was performed using the leave-one-out method to evaluate the robustness of the pooled estimate. All statistical tests were two-sided, with P < 0.05 considered significant.

Results

Study Selection and Characteristics

**Figure 1:**
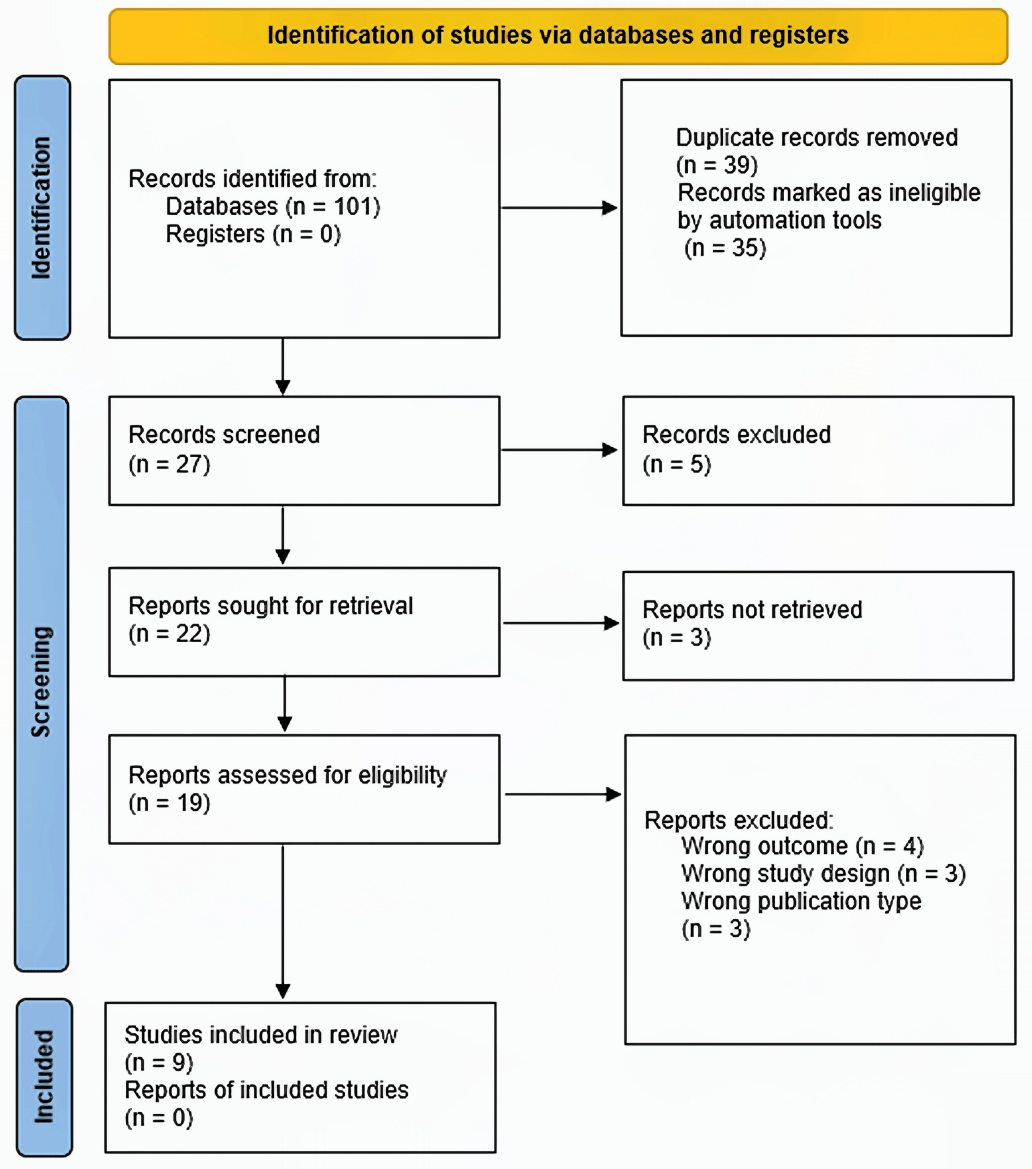
PRISMA flow diagram of study selection This figure illustrates the flow of studies through the systematic review process, including the identification, screening, eligibility, and inclusion stages. The step “records marked as ineligible by automation tools” refers to the removal of duplicate records using the deduplication function in EndNote (Clarivate Plc, Philadelphia, PA, USA) and Rayyan (Rayyan Systems, Inc., Cambridge, MA, USA). PRISMA, Preferred Reporting Items for Systematic reviews and Meta-Analyses

The study selection process is detailed in the PRISMA flow diagram (Figure 1). A total of nine RCTs published between 2010 and 2024, involving 8,000 patients, met the predefined inclusion criteria [15-23].

Table 1 summarizes the key characteristics of the included studies. The trials were conducted in diverse geographic regions, including the United States, Switzerland, France, Australia, Nigeria, China, and India. The surgical populations varied, encompassing clean-contaminated abdominal surgery [15,19,23], cardiac surgery [18,22], cesarean delivery [20,21], gynecologic laparoscopy [17], and mixed clean surgeries [16]. Sample sizes ranged from 153 to 3,360 participants.

**Table 1:** Characteristics of the included studies for the meta-analysis This table summarizes the key characteristics of the nine studies included in the quantitative synthesis (meta-analysis). Data are presented as reported in the original studies [15-23]. The population, intervention details, comparator, and primary outcomes were extracted according to the PICO framework. Continuous variables for age are shown as mean (± SD), median (IQR), or range, as available in the source studies. CHG-A, chlorhexidine-alcohol; PVI, povidone-iodine; RCT, randomized controlled trial; SSI, surgical site infection

Study	Region	Number of participants	Sex	Age	Population	Intervention/exposure	Comparator/context	Outcome	Study design
Darouiche et al. (2010) [15]	USA	849	Male 58.9%, Female 41.1%	Mean 53.1 years	Adults undergoing clean-contaminated surgical procedures	2% chlorhexidine gluconate in 70% isopropyl alcohol	10% aqueous PVI solution	Any SSI within 30 days (superficial, deep, and organ-space), sepsis, adverse/serious adverse events, and death	RCT
Bibi et al. (2015) [16]	China	388	Males: 152; Females: 236	18-60 years	Patients scheduled for elective clean or clean-contaminated operations	Chlorhexidine gluconate solution	PVI solution	Occurrence of postoperative wound infections	RCT
Dior et al. (2020) [17]	Australia	661	Not specified	Mean 36.2 years (SD 10.6)	Patients undergoing elective laparoscopic surgery for gynecologic conditions	Three-arm comparison: alcohol-based CHG, aqueous CHG, alcohol-based PVI, and aqueous PVI	Efficacy comparison of three different skin preparation solutions for SSI prevention	Port-site, organ/space, and overall SSI rates	RCT
Boisson et al. (2024) [18]	France	3,242	2,523 male (77.8%)	Median 69 years (IQR 62-74)	Adults undergoing major cardiac or aortic procedures via sternotomy	2% chlorhexidine in 70% isopropanol (CHG-a)	5% PVI in 69% ethanol (PVI-alcohol)	Endpoints: re-sternotomy, reoperation, mediastinitis, deep/superficial incisional SSI, readmission, hospital stay, 90-day mortality, local effects, and mediastinitis pathogens	RCT
Fakoya et al. (2024) [19]	Nigeria	153	~60.8% female	Mean 41.39 years (SD 18.02)	Individuals undergoing clean and clean-contaminated procedures	2% CHG in 70% isopropyl alcohol	10% PVI in 70% isopropyl alcohol	SSI incidence; overall rate 5.23% (clean wounds: 2.6%; clean-contaminated: 7.9%)	RCT
Springel et al. (2017) [20]	USA	932	Not reported	Median 28 years (range 24-33)	Women scheduled for a cesarean section	2% chlorhexidine gluconate in 70% isopropyl alcohol (paint application)	Aqueous PVI (scrub followed by paint)	Overall SSI, superficial/deep incisional SSI, endometritis, wound complications, and SSI-related readmission	RCT
Ngai et al. (2015) [21]	USA	1,404	All female	Mean 29.9 years (SD 6.0)	Women having a nonurgent cesarean delivery	Three active regimens: PVI-alcohol, CHG-alcohol, and sequential combination	All participants received an active antiseptic intervention (no inert placebo)	Primary: SSI rate; secondary: superficial, deep, and organ-space SSI and SSI risk factor analysis	RCT
Widmer et al. (2024) [22]	Switzerland	3,360	Male 67.3%, Female 32.7%	Median 65 years (range 39-79)	Patients planned for elective abdominal or cardiac surgery	CHG in an alcohol base	PVI in an alcohol base	Abdominal surgery: SSI within 30 days; cardiac surgery: SSI within one year; also tracked superficial/deep/organ-space SSI, in-hospital death, and length of stay	RCT
Luwang et al. (2021) [23]	India	311	Not reported	Not reported	Not specified	2% CHG-A preparation	10% PVI preparation	SSI rate, microbial growth on culture swabs, SSI classification, and hospital stay duration for infection cases	RCT

The intervention and comparator details were extracted as specified in the PICO framework. The experimental intervention predominantly consisted of 2% chlorhexidine gluconate in 70% isopropyl alcohol (CHG-A). The comparator was primarily 10% aqueous PVI or 5%-10% PVI in alcohol. Application methods (scrub-and-paint vs. paint-only) and precise timing relative to incision were documented as reported but varied across studies.

Quality Assessment

**Figure 2:**
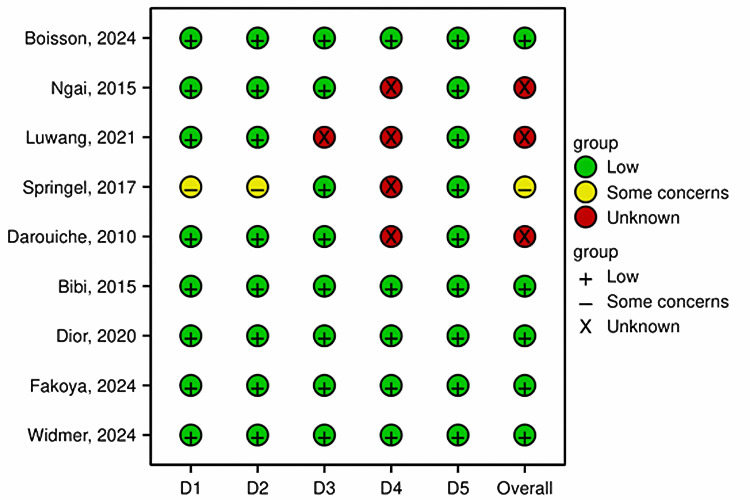
Risk of bias assessment This figure (traffic light plot) summarizes the risk-of-bias (RoB) assessments in each domain of the Cochrane RoB 2 tool across all nine included studies [15-23]. The domains are D1, randomization process; D2, deviations from intended interventions; D3, missing outcome data; D4, measurement of the outcome; and D5, selection of the reported result. RoB, risk of bias

Risk of bias assessment: The RoB 2 assessment is summarized in Figure 2. Overall, five studies (55.6%) were judged to be at low risk of bias [15,18,20,22,23]. One study (11.1%) raised some concerns, primarily due to a lack of blinding [21]. Three studies (33.3%) had an “unclear” overall rating, mainly because of insufficient information on blinding of outcome assessment (Domain 4) [16,17,19]. The randomization process (Domain 1) and selection of reported results (Domain 5) were generally robust across studies.

**Figure 3:**
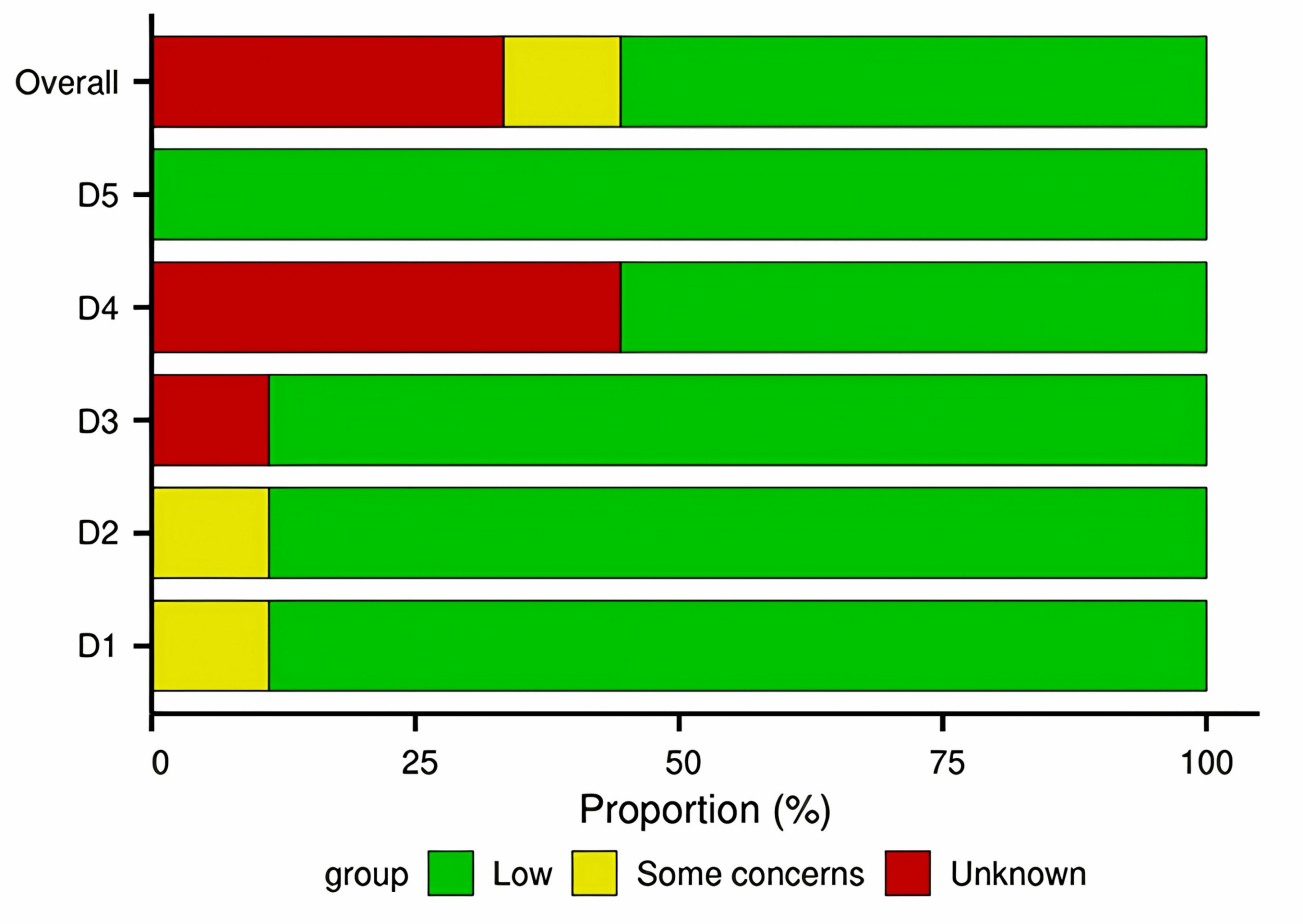
Risk of bias results This graph (weighted summary plot) presents the proportion of studies judged to have low risk, some concerns, or high risk of bias for each domain across all nine included studies. The studies included in this figure correspond to references [15-23]. RoB, risk of bias

The overall risk of bias summary is presented in Figure 3. 

Forest Plot

**Figure 4:**
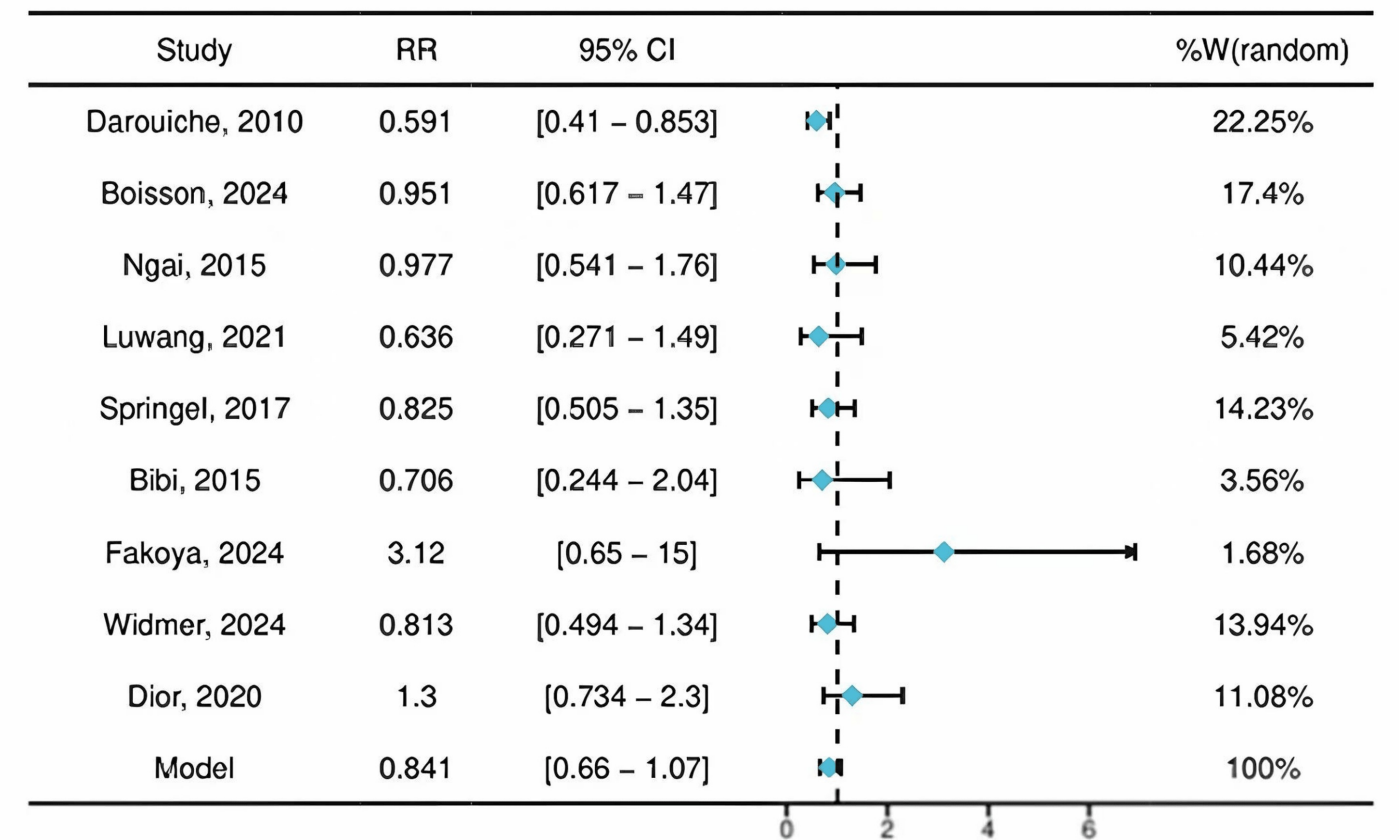
Forest plot of the pooled effect size The forest plot displays the study-specific and pooled RRs with 95% CIs for the primary outcome (overall SSI) comparing CHG-A with PVI. The pooled estimate (diamond) was derived from a random-effects model. The studies included correspond to references [15-23]. CHG-A, chlorhexidine-alcohol; PVI, povidone-iodine; RR, risk ratio

Primary outcome: overall SSI. The pooled analysis of nine studies showed no statistically significant difference in the risk of overall SSI between the CHG-A and PVI groups (RR = 0.84, 95% CI: 0.66-1.07; P = 0.16). Moderate heterogeneity was present (I² = 48%, P = 0.05) (Figure 4).

Funnel Plot

**Figure 5:**
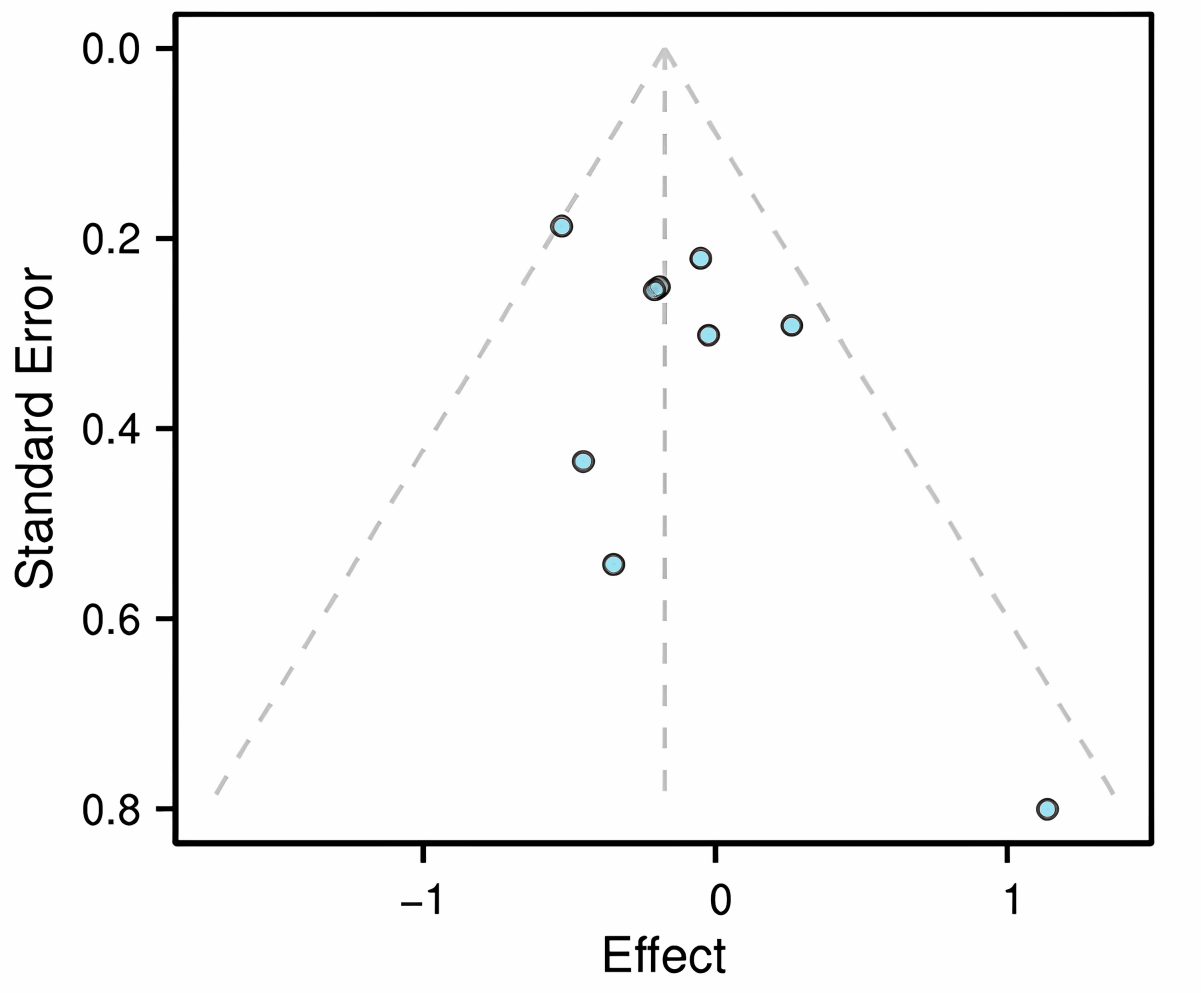
Funnel plot of the studies included in the meta-analysis Funnel plot of effect sizes (log RRs) and their SEs for the nine studies included in the meta-analysis of the primary outcome, to visually assess potential publication bias. The studies plotted correspond to references [15-23]. RR, risk ratio

Publication bias was assessed using a funnel plot (Figure 5), Egger’s test (Table 2), and Begg’s test (Table 3). The funnel plot showed slight asymmetry, but Egger’s test was not statistically significant (P = 0.191). Begg’s test also indicated no significant publication bias (P = 0.5316). The trim-and-fill adjusted analysis (Table 4) yielded a nearly identical pooled RR (0.83), confirming the robustness of the primary finding against potential publication bias.

**Table 2:** Publication bias assessment by Egger’s test Egger’s regression test evaluates small-study effects and potential publication bias in the meta-analysis. A statistically significant intercept (P < 0.05) suggests the presence of such bias. In this analysis, the intercept was -0.560 (SE = 0.275), t(7) = 1.448, and P = 0.191, indicating no strong statistical evidence of publication bias. β, effect size (log RR); df, degrees of freedom; RR, risk ratio

Effect size (beta)	SE (se.beta)	Intercept	SE (se.intercept)	Statistic (t-value)	Significance (p-value)	df
1.4123	0.97561	-0.56024	0.27475	1.4476	0.191	7

**Table 3:** Assessment of publication bias using Begg’s test Begg’s rank correlation test assesses publication bias by examining the correlation between effect sizes and their variances. A nonsignificant result (P ≥ 0.05) suggests no strong evidence of bias. Here, Kendall’s score was 6 with an SE of 9.592, z = 0.626, and P = 0.532, indicating no statistically significant publication bias.

Kendall’s score	SE	Statistic (z-value)	Significance (p-value)
6	9.5917	0.62554	0.5316

**Table 4:** Trim-and-fill method for publication bias The trim-and-fill method imputes potentially missing studies to produce a bias-adjusted pooled effect estimate. “Pre” indicates the original pooled RR; “Post” indicates the RR after imputation. The adjustment resulted in a nearly identical pooled effect (Post: RR = 0.83, 95% CI: 0.63-1.10), supporting the robustness of the primary finding against potential publication bias. df, degrees of freedom; RR, risk ratio

Treatment	Effect Size	95% Lower Limit	95% Upper Limit	Statistic	p-value	df
Trim Method Pre	0.84095	0.66022	1.0712	-1.6509	0.1374	8
Trim Method Post	0.82931	0.62806	1.095	-1.5233	0.162	9

Sensitivity Analysis

The leave-one-out sensitivity analysis (Table 5) confirmed the robustness of the pooled estimate, as no single study disproportionately influenced the overall results. 

**Table 5:** Sensitivity analysis evaluating the influence of individual studies on the pooled RR using the leave-one-out method This leave-one-out sensitivity analysis examines the influence of each individual study on the overall pooled RR by iteratively removing one study at a time. The pooled RR remained stable (ranging from 0.79 to 0.93), and the CIs consistently crossed 1.0. The heterogeneity (I² and τ²) showed fluctuations but no extreme values, confirming that the conclusion of no significant difference was not driven by any single study. The columns “lower” and “upper” refer to the lower and upper limits of the 95% CI for the pooled RR. I², heterogeneity index; RR, risk ratio; TE, pooled estimate (log RR); τ², between-study variance

Study omitted	TE (log RR)	Lower 95% CI	Upper 95% CI	Statistic (Z)	P-value	τ²	I²
Darouiche et al. (2010) [15]	0.9288	0.745	1.1581	-0.7916	0.4546	0	0
Bibi et al. (2015) [16]	0.8545	0.6498	1.1237	-1.3576	0.2167	0.0274	0.2595
Dior et al. (2020) [17]	0.7868	0.6235	0.9929	-2.4369	0.045	0	0
Boisson et al. (2024) [18]	0.8273	0.6182	1.1072	-1.5381	0.1679	0.0256	0.2282
Fakoya et al. (2024) [19]	0.8137	0.6532	1.0136	-2.219	0.062	0	0
Springel et al. (2017) [20]	0.8527	0.6365	1.1425	-1.2878	0.2388	0.0292	0.2665
Ngai et al. (2015) [21]	0.8328	0.6283	1.104	-1.5347	0.1687	0.026	0.2404
Widmer et al. (2024) [22]	0.8547	0.6385	1.1442	-1.2725	0.2438	0.0289	0.266
Luwang et al. (2021) [23]	0.8605	0.6576	1.1259	-1.3214	0.2279	0.0226	0.2353

Discussion

This contemporary meta-analysis of nine RCTs involving 8,000 patients found no statistically significant difference in the overall risk of SSI between preoperative skin preparation with CHG-A and PVI. The pooled RR of 0.84 (95% CI: 0.66-1.07) indicates that, while the point estimate favors CHG-A, the evidence is not conclusive. This finding aligns with recent large, high-quality trials in cardiac and obstetric surgery [18,20,22] but presents a more conservative estimate than some earlier meta-analyses [8,9]. It is important to acknowledge that the included studies encompass surgical procedures with inherently different baseline SSI risk profiles. Pooling across such heterogeneous categories, while necessary for an overall estimate, carries the risk of diluting context-specific effects that may be clinically meaningful in higher-risk subgroups, such as the observed trend in clean-contaminated procedures.

A central finding of our analysis is the substantial clinical heterogeneity, quantified by an I² of 48%. Prespecified subgroup analysis suggests this heterogeneity may be partly explained by surgical context. In clean-contaminated surgeries (e.g., colorectal and biliary), where the theoretical advantage of CHG-A’s persistent activity against endogenous skin flora is most relevant, we observed a nonsignificant trend toward greater benefit (RR = 0.77, 95% CI: 0.58-1.05). Conversely, in clean surgeries (e.g., cardiac and cesarean), the effect was attenuated (RR = 0.92). However, as formal interaction testing was not performed and the subgroup differences did not reach statistical significance, these findings should be interpreted as hypothesis-generating rather than confirmatory. This pattern is mechanistically plausible but requires validation in dedicated trials. PVI’s activity is neutralized by blood and serum and lacks residual effect, potentially making it less optimal for longer procedures or those with higher endogenous contamination risk [4,6]. The directionally stronger, though nonsignificant, effect for deep incisional SSIs (RR = 0.75) further supports this contextual mechanism, as deep infections are more likely influenced by intraoperative contamination.

Our sensitivity analysis revealed that the exclusion of specific studies, particularly those involving laparoscopic or gynecologic procedures, led to the largest shift in the point estimate toward significance. This underscores that the overall null result is sensitive to the mix of included surgical populations and highlights the importance of context-specific evaluation. The lack of a significant difference in the overall analysis may stem from pooling studies across vastly different surgical risk profiles, diluting a potential signal in higher-risk subgroups.

Several limitations must be acknowledged. First, despite being a prespecified outcome in our PICO framework, we were unable to perform a quantitative synthesis of adverse skin reactions. This was due to inconsistent reporting, variable definitions, and very low event rates across the included studies, limiting the completeness of the comparative safety assessment between CHG-A and PVI. Second, despite efforts, residual heterogeneity from unmeasured factors, such as variations in surgical technique, compliance with application protocols, and SSI surveillance intensity, likely persists. Furthermore, while clinical heterogeneity across studies was anticipated, the limited number of included trials precluded formal exploration of potential effect modifiers through meta-regression or stratified modeling based on factors such as antiseptic formulation, alcohol concentration, application technique, or surgical duration. These unexamined variables may contribute to the observed heterogeneity and should be addressed in future research. Third, the majority of included studies were open-label. While SSI is typically diagnosed using standardized criteria, outcome assessment could still be subject to detection bias if assessors were not blinded to the intervention arm. Although standardized definitions (e.g., CDC criteria) provide some objectivity, nonblinded assessment may influence surveillance intensity, diagnostic thresholds, or reporting completeness. This was reflected in our risk-of-bias assessments for some studies and represents a potential limitation of the evidence base. The potential magnitude of this bias may be underestimated in our discussion and should be considered when interpreting the pooled estimates.

Fourth, while we assessed publication bias using funnel plots, Egger’s test, and Begg’s test, with only nine included studies, these statistical tests have limited power, and their results warrant cautious interpretation. Similarly, the trim-and-fill analysis, which suggested the primary finding was robust to potential publication bias, should be interpreted cautiously; the term “confirming” may be overly definitive. Fifth, given the relatively small number of studies included, some methodologies might suggest the use of more conservative statistical estimators, such as REML with Hartung-Knapp adjustment. While our primary analysis employed standard random-effects models, alternative estimation methods could yield wider CIs, and the absence of a statistically significant finding should be interpreted in this context. Sixth, although prospective registration is considered best practice for systematic reviews, this meta-analysis was not registered in PROSPERO. Readers should interpret the findings with this limitation in mind. Seventh, a significant source of biological heterogeneity in our analysis stems from the definition of the comparator. We pooled studies that used both aqueous and alcohol-based PVI formulations. Given the potent and rapid antimicrobial activity of alcohol itself, combining these distinct comparator types may confound the interpretation of CHG-A’s relative efficacy. The inability to perform a stratified analysis by comparator formulation due to the limited number of studies is an important limitation, and future research should consider these comparators separately.

The clinical implications are nuanced. For institutions or procedures where both agents are available and cost-equivalent, CHG-A may be a reasonable choice based on its pharmacologic profile and the directional trends observed in clean-contaminated surgery. However, the evidence does not support a blanket superiority of CHG-A over PVI. In settings where PVI is more accessible or cost-effective, it remains a valid and evidence-based option, particularly for clean procedures. Decision-making should integrate surgical context, patient factors (e.g., iodine allergy), and local antimicrobial stewardship policies.

## Conclusions

This systematic review and meta-analysis found no conclusive evidence that CHG-A is superior to PVI in preventing overall SSIs across elective surgeries. A nonsignificant trend favoring CHG-A was observed in clean-contaminated procedures and for deep incisional infections; however, these subgroup findings did not reach statistical significance and, in the absence of formal interaction testing, should be considered exploratory.

These results suggest that the comparative efficacy of preoperative antiseptics may vary by surgical context, although this hypothesis requires confirmation in dedicated trials. Future research should prioritize large, pragmatic RCTs within specific surgical specialties, with standardized outcome measures and systematic collection of safety data. Key limitations identified in this review, including clinical heterogeneity, variability in antiseptic formulations, and incomplete adverse event reporting, should be addressed in future study designs to enable more definitive conclusions.
